# An Improved SM2 Digital Signature Algorithm with High-Precision Timestamps for Trusted Metrological Data

**DOI:** 10.3390/s25164920

**Published:** 2025-08-09

**Authors:** Zhanshuo Cao, Boyong Gao, Xingchuang Xiong, Zilong Liu

**Affiliations:** 1 School of Information Engineering, China Jiliang University, Hangzhou 310018, China; pinocchioqaq@outlook.com (Z.C.); gaoby@cjlu.edu.cn (B.G.); 2 Center for Metrology Scientific Data, National Institute of Metrology, Beijing 100029, China; xiongxch@nim.ac.cn; 3 National Metrology Data Center, Beijing 100029, China; 4 Key Laboratory of Metrology Digitalization and Digital Metrology, State Administration for Market Regulation, Beijing 100029, China

**Keywords:** elliptic curve cryptography, digital signature, high-precision timestamp, metrological data, data traceability

## Abstract

**Highlights:**

**What are the main findings?**

- A modified SM2 digital signature algorithm is proposed, effectively enhancing the security and traceability of metrological data.
- By optimizing the signature structure and integrating high-precision time information, the algorithm significantly improves computational efficiency while ensuring data integrity and non-repudiation.

**What are the implications of the main findings?**

- It effectively mitigates security risks arising from random number dependency and replay attacks, providing a reliable and efficient security solution for scenarios requiring high trust and stringent time-based authentication.
- The improved algorithm offers high computational efficiency and strong traceability for data, such as digital documents, making it suitable for application scenarios with high security requirements.

**Abstract:**

With the advancement of modern technologies, the digitization of metering data has significantly improved the efficiency and accuracy of data collection, analysis, and management. However, the growing prevalence of data tampering techniques has raised serious concerns regarding the trustworthiness and integrity of such data. To address this challenge, this study proposes an improved SM2 digital signature algorithm enhanced with high-precision time information to strengthen the reliability of metering data. The proposed algorithm incorporates high-precision timestamps into the signature generation and verification processes, while optimizing the structure of the signature algorithm—particularly the modular inversion operation—to reduce computational costs. Experimental results demonstrate that the improved algorithm not only significantly enhances signature generation efficiency but also improves temporal validity and security by leveraging high-precision time information. It effectively mitigates risks associated with random number dependency and replay attacks, offering a secure and efficient solution for trustworthy metering data verification.

## 1. Introduction

Metrology serves as a vital foundation supporting the construction of national integrated strategic systems and capabilities, with scientific data in this field playing a crucial role as fundamental data standards [1,2]. Ensuring the trustworthiness of metrological scientific data is essential for the effective establishment of standard reference data and for enabling national metrology standards to fulfill their core function in data sharing processes [3,4,5]. With the advancement of technologies such as the Internet of Things (IoT), edge computing, and intelligent manufacturing, metrology systems are evolving toward digitalization and networking, thereby making data collection, transmission, and storage more efficient and convenient [6,7]. However, this digital transformation also introduces new challenges: data may be vulnerable to malicious tampering, forgery, or replay attacks during transmission, which pose significant threats to the integrity and reliability of metrological data [8,9].

To safeguard the security and traceability of metrological data, digital signature technologies have been widely applied in scenarios requiring tamper-proofing and identity authentication [10,11,12]. Among them, the SM2 algorithm, as an elliptic curve public key cryptosystem independently developed in China, has been extensively used in critical information infrastructure due to its high security strength and computational efficiency [13,14,15,16]. Nevertheless, the traditional SM2 algorithm still faces certain challenges in practical applications [17], such as security risks arising from its dependence on random numbers and the lack of effective binding between data and its generation time, limiting its suitability for high-trust metrological scenarios.

In response to these issues, this paper proposes an improved digital signature algorithm inspired by SM2 and based on high-precision timestamps. By introducing high-precision timestamps generated by a trusted time source, the proposed algorithm enhances the temporal consistency and non-repudiation of signature data, thereby strengthening the system’s resistance to replay attacks and data forgery. Furthermore, the signature process is structurally optimized to improve computational efficiency and meet the dual demands of real-time performance and security in large-scale metrology systems. Experimental results demonstrate that the proposed method ensures metrological data security while offering promising prospects for practical application and broader adoption.

## 2. Related Work

In recent years, Elliptic Curve Cryptography (ECC)-based digital signature algorithms have been widely adopted in the fields of the Internet of Things (IoT) and data security due to their high level of security and computational efficiency [18]. To address the computational complexity and performance bottlenecks caused by modular inversion in traditional ECDSA on resource-constrained devices, Yang et al. [19] proposed a lightweight NOMOP-ECDSA algorithm. This algorithm replaces the computationally intensive modular inversion operation with more efficient basic operations, significantly reducing resource consumption. It is particularly suitable for low-power devices, such as smart cards, and has been further extended into a three-phase mutual authentication protocol supporting authorization, authentication, and key updates, with strong resistance to forgery and human-in-the-middle attacks.

To mitigate the risks of signature reuse and private key leakage, Puthiyidam et al. [20] introduced the Temporal ECDSA algorithm. By incorporating timestamps and signature masking into the traditional signing structure, this method effectively prevents signature reuse from the source. The algorithm has been successfully embedded into the lightweight MQTT communication protocol, demonstrating its feasibility and superiority on low-resource devices.

Extensive research has been conducted on the improvement and application of the SM2 signature algorithm. Shao et al. [21] integrated SM2 into power information collection systems, combining it with the SM3 hash function, optimized random number generation, and timestamp mechanisms to construct a secure communication protocol for bidirectional authentication in power IoT environments, thereby enhancing the system’s resistance to replay and tampering attacks. Wu et al. [22] proposed a lightweight security authentication scheme based on SM2 for smart grids. By optimizing the SM2 signature algorithm and incorporating symmetric encryption techniques, the scheme achieves efficient key agreement and identity authentication while significantly reducing computational and communication overhead.

In terms of hardware implementation and computational optimization, Chen et al. [23] designed a highly efficient parallel modular multiplication scheme based on the Karatsuba algorithm and a reusable multiplier structure. This scheme was implemented on an FPGA platform, achieving low resource consumption and high-speed performance suitable for embedded security scenarios. Xu et al. [24] proposed an SM2 collaborative signature system that employs private key slicing, signature preprocessing optimization, and PIN-based identity authentication mechanisms. This design enables an efficient collaborative signature solution for smart mobile terminals, enhancing both key management security and signing efficiency. Meng et al. [25] presented an improved SM2-based algorithm, extending the key length to 512 bits. By introducing multi-threaded parallel computing and homomorphic encryption, the algorithm significantly improves both security and computational efficiency, demonstrating strong decryption performance and resistance to attacks in power and cloud environments. Zhu et al. [26] addressed the limited resources of IoT terminal devices by proposing an optimization scheme based on improved Montgomery multiplication and polynomial segmentation. By leveraging parallel computing and SIMD instruction sets, their approach enhances the efficiency of SM2 modular and scalar multiplication, yielding substantial performance improvements on IoT devices.

Although existing studies have introduced time-related features into digital signature algorithms to enhance data timeliness and non-repudiation, most of them rely on low-precision time information [20,21], which is insufficient for meeting critical security requirements such as high timeliness, strong non-repudiation, and full-chain traceability in the generation, transmission, and sharing of metering data. Therefore, this paper proposes a digital signature algorithm, improved from SM2 and based on high-precision timestamps. Building upon the original security properties of SM2, the proposed scheme incorporates high-precision timestamps generated by a trusted time source and structurally reconstructs the signature process. This enhancement significantly improves the system’s resistance to replay attacks and ensures end-to-end traceability during metering data authentication and transmission. The proposed approach is expected to provide a trustworthy, traceable, and efficient security mechanism for future digital metering systems.

## 3. Prior Knowledge

### 3.1. Elliptic Curve Cryptography

ECC is a public-key cryptographic scheme based on the mathematical structure of elliptic curves. The concept was independently proposed in 1985 by mathematicians Neil Koblitz [27] and Victor Miller [28]. ECC is built upon the algebraic structure of elliptic curves, with its core mathematical foundation being the elliptic curve group defined over finite fields. Elliptic curves are typically expressed in the Weierstrass standard form, which is algebraically represented as follows:

(1) $$E:{\;y}^{2}=x^{3}+ax+b\;(mod\;p)$$

The elliptic curve is defined over a finite field *F_p_*, where *p* is a prime modulus, and $a,b\in F_{p}$ are the curve’s coefficient parameters, which together determine the shape and properties of the curve.

**Figure 1:**
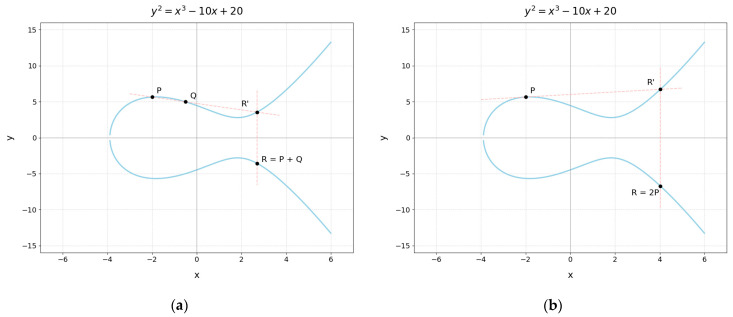
(**a**) Point addition on an elliptic curve. (**b**) Point doubling on an elliptic curve.

Mathematical operations on elliptic curves, such as point addition, can be intuitively understood as follows: given any two points on the curve, a straight line is drawn through them. This line will typically intersect the curve at a third point, denoted as *R*′. Reflecting this point across the x-axis yields a new point *R*, which is defined as the sum of the two original points, i.e., R=P+Q. Similarly, if a tangent line is drawn at a single point *P* on the curve, it will intersect the curve at another point. Reflecting this intersection point across the x-axis gives a new point *R*, which defines the point doubling operation, denoted as R=2P. Figure 1 illustrates examples of point addition and doubling on an elliptic curve.

The security of ECC relies on the computational intractability of the Elliptic Curve Discrete Logarithm Problem (ECDLP) [29,30]. Specifically, given a base point *G* and a point K=k⋅G, computing *K* from a known scalar *k* and point *G* is a forward operation of scalar multiplication on the elliptic curve, which can be performed efficiently. However, the inverse problem—determining the scalar *k* given *G* and *K*—is computationally infeasible. This one-way property arises from the discrete nature of elliptic curves over finite fields. Currently, no efficient algorithm is known that can solve the ECDLP within a reasonable amount of time. The SM2 algorithm, as well as the widely adopted international ECDSA [31] digital signature algorithm, relies on the computational hardness of ECDLP as its fundamental security basis. This computational difficulty provides the foundational security guarantee for ECC. However, it is important to note that not all elliptic curves in ECC offer the same level of security. In particular, certain curves can become highly vulnerable when insecure random numbers are used [32]. Therefore, selecting elliptic curves that have been thoroughly vetted and possess strong security properties is essential to ensuring the overall security of ECC.

### 3.2. SM2 Digital Signature Algorithm

The SM2 digital signature algorithm, developed by the State Cryptography Administration of China, is a public key signature scheme based on elliptic curve cryptography. It was adopted as an international standard in 2018 under the designation ISO/IEC 14888-3/AMD1 [33]. Similar to other signature algorithms, SM2 employs a private key to generate the signature and a corresponding public key to verify its validity. However, it differs from international standards in terms of algorithmic procedures, key agreement mechanisms, and recommended elliptic curve parameters (see Table 1), adopting a design with enhanced security features.

To strengthen the binding between the signature and the signer’s identity and improve resistance against attacks, SM2 introduces a user identifier denoted as *Z* prior to signature generation. This identifier is concatenated with the original message and then hashed. The hashing is performed using the SM3 algorithm, which is also specified by the State Cryptography Administration and designed specifically for China’s commercial cryptographic framework. In terms of both security and performance, SM3 is comparable to SHA-256 [34].

## 4. Methods

### 4.1. The Improved Algorithm T_SM2

To meet the high standards of traceability, security, and temporal relevance required for data—particularly electronic documents—in the field of metrology, this paper proposes an improved digital signature algorithm, T_SM2. Built upon the traditional SM2 signature scheme, T_SM2 integrates high-precision timestamping and optimizes the signature process. By employing high-accuracy time synchronization devices, the algorithm generates nanosecond-level timestamps to provide precise and trustworthy temporal markers for the signed data. These timestamps are not only embedded within the signed message but also directly involved in the signature computation, thereby establishing a strong temporal binding. This mechanism effectively mitigates risks such as timestamp forgery and replay attacks using historical signatures. During verification, the recipient can evaluate the signature’s validity and timeliness by comparing the embedded timestamp with the current system time, thus enhancing the assurance of data authenticity, integrity, and traceability. Table 2 presents the parameters used in the proposed scheme.

### 4.2. High-Precision Timestamp

High-precision timestamps are a critical parameter for ensuring data traceability and security. Their core value lies in providing digital objects with a tamper-resistant time reference featuring nanosecond-level accuracy. In the proposed algorithm, timestamp generation relies on highly stable time–frequency references delivered by GNSS common-view receivers [35]. Under the condition that multiple stations are simultaneously locked onto the same satellite, inter-station time synchronization accuracy better than 5 nanoseconds can be achieved, enabling the timestamps to be traceable to Coordinated Universal Time (UTC).

The receiver integrates a high-stability crystal oscillator, which dynamically compensates for frequency drift through a disciplining algorithm. Even in the case of temporary satellite signal loss, it can maintain high timing stability: the short-term frequency stability is better than $3\times 10^{-12}/s$, phase noise is as low as −155 dbc/Hz@1 kHz, and the long-term holdover performance achieves a time deviation of less than 10 μs over 24 h, ensuring the continuity and reliability of time information output.

**Figure 2:**
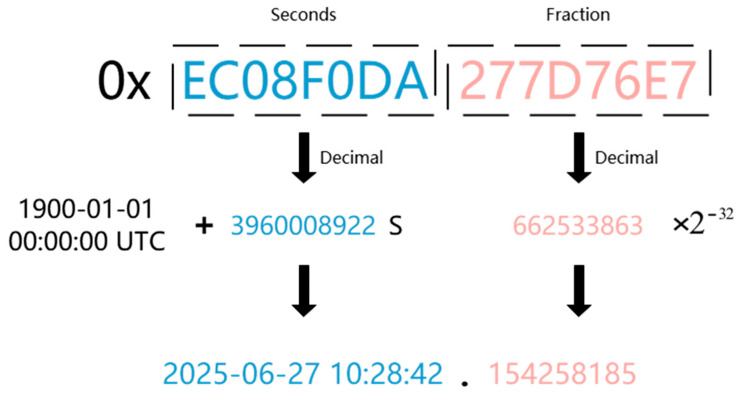
High-precision timestamp structure.

To unify the time format, the device outputs time information encoded using a 64-bit high-precision timestamp structure, as shown in Figure 2. The first 32 bits represent the number of whole seconds elapsed since 1 January 1900, 00:00:00, while the remaining 32 bits denote the fractional part of the current second as an unsigned integer. This structure essentially provides a binary high-precision representation of time relative to UTC, offering nanosecond-level accuracy and ensuring cross-system compatibility and verifiability of time identification.

In the algorithm, the timestamp *T* serves as a core participating factor in both the signing and verification processes, directly influencing the computation of the signature *sig*. This design embeds the timestamp as an intrinsic attribute of the signed data, such that any tampering with *T* results in signature verification failure. Consequently, it establishes a strong binding between the data and its associated time, endowing the digital signature with non-repudiation, time verifiability, and full lifecycle traceability.

### 4.3. Signature Generation Stage

**Figure 3:**
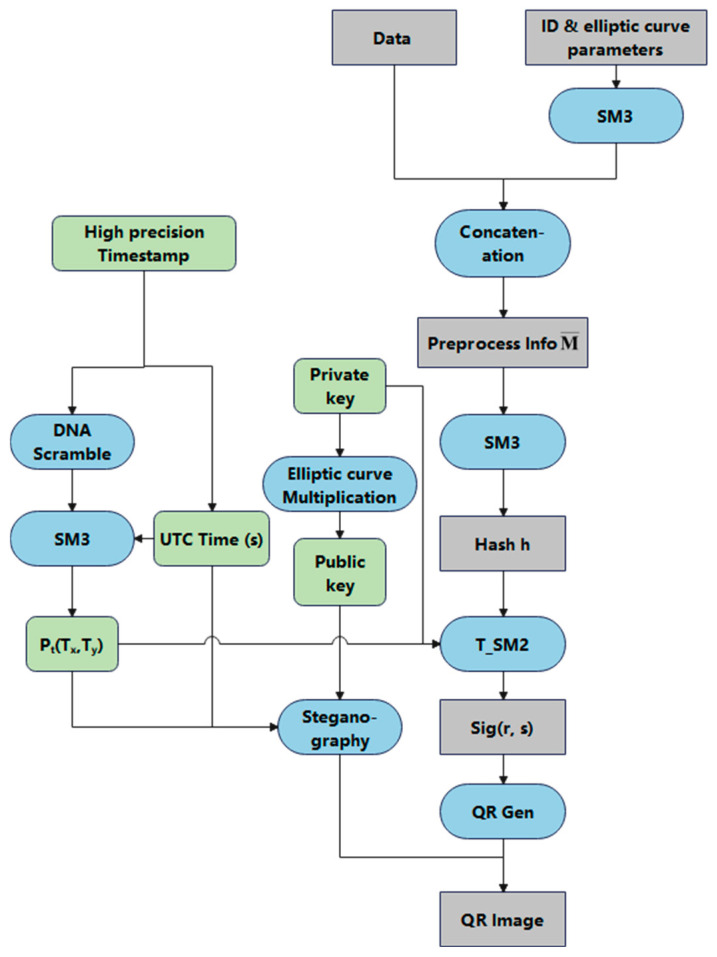
T_SM2 algorithm signature generation process.

The signature generation process is illustrated in Figure 3. Unlike the traditional SM2 signature scheme, the proposed method introduces timestamp information *T* as a dynamic source of randomness. After undergoing DNA encoding and scrambling, this information is incorporated into the computation of the digital signature (*r*, *s*), thereby enhancing the dynamism of the signature and reducing the algorithm’s dependence on the random number *k*.

Due to the inherently monotonically increasing nature of timestamp data, directly using it may introduce predictability risks. If an attacker can predict the trend of the timestamp, they could potentially infer key parameters used in the signature generation through analytical attacks, thereby compromising security. Therefore, the direct application of traditional timestamps may pose significant security vulnerabilities. To address this issue, this study proposes a DNA encoding and scrambling technique. By transforming timestamp data into a more complex and highly uncertain form, DNA encoding effectively increases the entropy and unpredictability of the timestamp, thereby reducing its predictability and enhancing security. The scrambled timestamp is then hashed and embedded into the signature generation process, thereby improving the overall security of the signature output.

DNA encoding is a sequence mapping method inspired by bioinformatics [36], where four nucleotides—adenine (A), thymine (T), cytosine (C), and guanine (G)—are used to represent binary data. Each nucleotide can be encoded using a two-bit binary representation, such as A–00, C–01, G–10, and T–11. Among them, A pairs with T, and C pairs with G, following the Watson–Crick complementary pairing principle. Out of 24 possible encoding rules, only 8 conform to the Watson–Crick complementarity [37], as shown in Table 3.

Meanwhile, T_SM2 optimizes the modular inversion operation in the traditional SM2 signature process, thereby reducing computational overhead. The entire signing phase consists of the following four steps.

Step 1: A private key $d\in \left[1,n-2\right]$ is generated using a random number generator, and the corresponding public key is computed as P=d⋅G through elliptic curve point multiplication. An identifier *ID* is then extracted from the electronic document or related data, and the hash value *Z* is computed using the SM3 hash function as Z=SM3(len(ID)||ID||a||b||G||P). Subsequently, the message *M* is read and concatenated with *Z* to form the preprocessed message $\bar{M}=Z||M$.

Step 2: Based on the timestamp *T*, two dynamic components are generated: $T_{x}=SM3(T_{u})$ and $T_{y}=SM3(DNA(T))$, where *DNA(T)* refers to a DNA-based scrambling transformation of the timestamp. These components are concatenated to produce the dynamic factor $P_{t}=T_{x}||T_{y}$.

Step 3: The preprocessed message $\bar{M}$ is hashed using the SM3 function to obtain the digest $h=SM3\left(\bar{M}\right)$. A random integer $k\in \left[1,n-1\right]$ is then generated, and elliptic curve point multiplication yields the point $\left(x_{1},y_{1}\right)=k\cdot G$. The signature is computed in two parts, *r* and *s*, as follows:

(2) $$r=\left(h+x_{1}+T_{y}\right)\;mod\;n$$

(3) $$s=\left(k-\left(r+T_{x}\right)\cdot d\right)\;mod\;n$$

Step 4: The signature *sig* is encoded into a QR code image, and verification information, including the public key *P*, *T_y_*, and UTC time *T_u_*, is created. The verification information is embedded into the QR code image using LSB-based steganography to ensure that the information is invisible in the image but can be extracted. The modified image is then saved as the final signature file. The signing phase algorithm is shown in Algorithm 1.
**Algorithm 1**: T_SM2 algorithm signature generation processInput: Message *M*, identifier *ID*, timestamp *T*, elliptic curve parameters (*a*, *b*, *G*, *n*)Output: signed QR code imageGenerate private key $d\in \left[1,n-2\right]$, compute public key P=d⋅GZ=SM3(len(ID)||ID||a||b||G||P)$\bar{M}=Z||M$If *T* is null or invalid then  Return errorEnd if$T_{x}=SM3(T_{u})$, $T_{y}=SM3(DNA(T))$$P_{t}=T_{x}||T_{y}$$h=int(SM3\left(\bar{M}\right),16)$Repeat  Generate random $k\in \left[1,n-1\right]$  $\left(x_{1},y_{1}\right)=k\cdot G$  $r=\left(h+x_{1}+T_{y}\right)\;mod\;n$  If r=0 or r+k=n then    Continue (restart loop)  End if  $s=\left(k-\left(r+T_{x}\right)\cdot d\right)\;mod\;n$  If s=0 then    Continue (restart loop)  End ifUntil valid (*r*, *s*) found$sig=\left(r,s\right)$Encode *sig* into QR codeEmbed *P*, *T_y_*, *T_u_* into QR via LSB steganographySave as signed image

### 4.4. Signature Verification Stage

**Figure 4:**
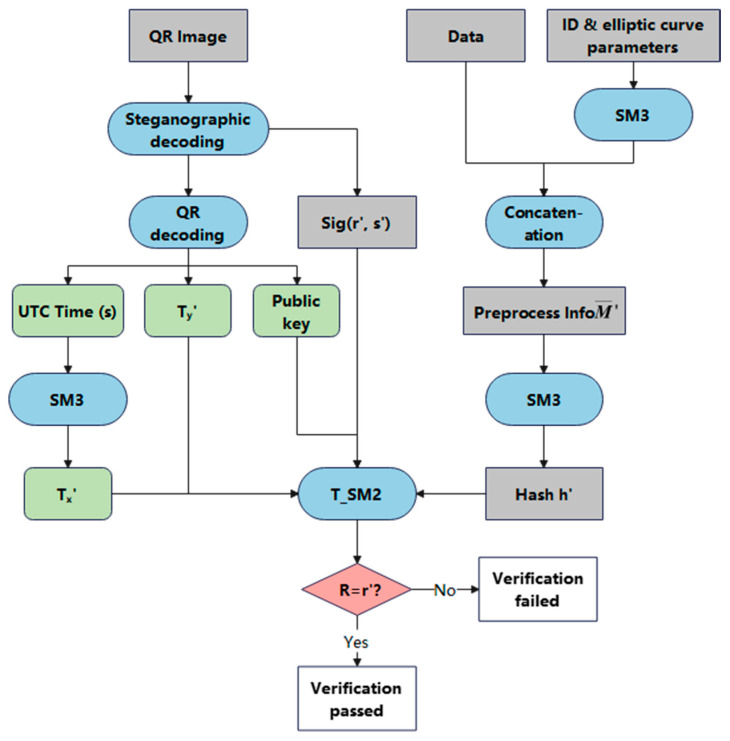
T_SM2 algorithm signature verification process.

The signature verification phase can be regarded as the reverse process of signature generation. The main task in this phase is to extract the verification information and digital signature from the QR code image embedded within the steganographic data, after receiving the electronically signed document. It then verifies the extracted timestamp and the validity of the digital signature. The signature verification process is illustrated in Figure 4.

Step 1: Open the QR code image containing steganographic information. Use steganographic decoding techniques to extract the public key *P*, dynamic factor *T_y_*′, and UTC time *T_u_*′. Then, scan the image to extract the embedded digital signature *sig*′.

Step 2: Convert *T_u_*′ to byte format, representing the number of seconds since 1900-01-01 00:00:00 UTC. Compute its SM3 hash to obtain *T_x_*′.

Step 3: Obtain the message *M*′ and the identifier *ID*. Use the SM3 hash function to generate the corresponding *Z*, and concatenate it with *M*′ to construct the message $\bar{M^{\prime }}=Z||M^{\prime }$. Hash $\bar{M^{\prime }}$ using SM3 to obtain $h^{\prime }=SM3(\bar{M^{\prime }})$. Decompose the signature *sig*′ to extract the signature components *r*′ and *s*′. Compute *t* using Equation (4):

(4) $$t=\left(r^{\prime }+T_{x}^{\prime }\right)\;mod\;n$$

Then, compute the elliptic curve point multiplication and addition to obtain point $\left({x_{1}}^{\prime },{y_{1}}^{\prime }\right),$ as shown in Equation (5):

(5) $$\left({x_{1}}^{\prime },{y_{1}}^{\prime }\right)=[s^{\prime }]\cdot G+[t]\cdot P$$

If the condition $\left[s^{\prime }\right]\cdot G=\left[t\right]\cdot P$ holds, point addition is not applicable. In this case, point doubling should be performed to compute: $\left({x_{1}}^{\prime },{y_{1}}^{\prime }\right)=[2]([s^{\prime }]\cdot G)$.

Step 4: Extract the x-coordinate *x*_1_′ and compute *R* using Equation (6):

(6) $$R=\left(h+{x_{1}}^{\prime }+T_{y}^{\prime }\right)\;mod\;n$$

Compare *R* with *r*′. If $R=r^{\prime }$, the signature is valid; otherwise, verification fails. The algorithm for the verification phase is shown in Algorithm 2.
**Algorithm 2**: T_SM2 algorithm signature verification processInput: Message *M*′, identifier *ID* QR code image, public key *P*, elliptic curve parameters (*a*, *b*, *G*, *n*)Output: Signature validity result (Accept or Reject)Use LSB decoding to extract steganographic data from the QR code image:  Retrieve signature $sig\;=\;(r^{\prime },\;s^{\prime })$  Extract public key *P*, dynamic factor *T_y_*′, and UTC timestamp *T_u_*′  If any of the extracted data is missing or malformed then    Return Reject  End ifIf *T_u_*′ is null or invalid then  Return RejectEnd ifConvert *T_u_*′ to seconds since 1900-01-01 00:00:00 UTC${T_{x}}^{\prime }=SM3({T_{u}}^{\prime })$$P_{t}={T_{x}}^{\prime }||{T_{y}}^{\prime }$Z=SM3(len(ID)||ID||a||b||G||P)$\bar{M^{\prime }}=Z||M^{\prime }$$h=int(SM3\left(\bar{M^{\prime }}\right),16)$$t=\left(r^{\prime }+T_{x}^{\prime }\right)\;mod\;n$If $\left[s^{\prime }\right]\cdot G==\left[t\right]\cdot P$ then  $\left({x_{1}}^{\prime },{y_{1}}^{\prime }\right)=[2]([s^{\prime }]\cdot G)$Else  $\left({x_{1}}^{\prime },{y_{1}}^{\prime }\right)=[s^{\prime }]\cdot G+[t]\cdot P$  Convert to affine coordinates if neededEnd if$R=\left(h+{x_{1}}^{\prime }+T_{y}^{\prime }\right)\;mod\;n$If $R==r^{\prime }$ then  Return AcceptElse  Return RejectEnd if

## 5. Experiment

### 5.1. Experimental Conditions

The security and efficiency of the proposed algorithm are evaluated in this section. The experimental conditions are shown in Table 4.

### 5.2. Security Analysis

#### 5.2.1. Comparison and Analysis of Signature Algorithms

As shown in Table 5, the T_SM2 algorithm, like ECDSA and SM2, is based on the ECDLP. Compared to RSA, which relies on the integer factorization problem, T_SM2 offers a significant advantage in terms of key length: to achieve the same level of security, T_SM2 requires only a 256-bit key, whereas RSA requires a 3072-bit key. This results in improved computational efficiency and resource utilization.

In the process of generating digital signatures, most algorithms utilize hash functions to produce fixed-length message digests. This not only significantly reduces the computational load of the signing process but also ensures data integrity and resistance to tampering. Therefore, the collision resistance and computational performance of the hash function are critical considerations. To address these requirements, the T_SM2 algorithm adopts the Chinese national cryptographic hash function SM3, which offers collision resistance and computational performance comparable to the widely used SHA-256, thereby ensuring strong security and good compatibility

In the subsequent stages, T_SM2 further supports user identity binding and timestamp authentication, and it reduces reliance on random number generation. These features further enhance its security and applicability in scenarios requiring high identity assurance and strict time sensitivity.

#### 5.2.2. Attack Resistance Analysis

In elliptic curve-based digital signature algorithms, the random number used during the signing process is a critical parameter that directly determines both the uniqueness and security of the signature. If the same random number is reused, it can lead to serious risks of private key leakage. Both ECDSA and the standard SM2 are susceptible to private key recovery attacks. Taking ECDSA as an example, its signature consists of a pair of values (*r*, *s*), where:

(7) $$r={\left(k\cdot G\right)}_{x}\mathrm{mod}n,\;\;s=k^{-1}\left(h+\mathrm{d}\cdot r\right)\mathrm{mod}n$$

If the same random nonce *k* is used to sign two different messages, the resulting signatures will share the same *r* value, forming two signature pairs (*r*, *s*_1_) and (*r*, *s*_2_). By subtracting *s*_2_ from *s*_1_, the random nonce *k* can be derived (Equation (8)):

(8) $$s_{1}-s_{2}=k^{-1}\left(h_{1}-h_{2}\right)\mathrm{mod}n\to k=\left(h_{1}-h_{2}\right)\cdot {\left(s_{1}-s_{2}\right)}^{-1}\mathrm{mod}n$$

Once the nonce *k* is recovered by an attacker, it can be substituted into the signature equation to solve for the private key *d*.

This vulnerability has led to severe consequences in real-world systems. For example, in 2010, Sony’s PlayStation 3 suffered an ECDSA private key leak due to the use of a fixed *k* [38]. Similarly, in 2013, a flaw in the random number generator on the Android platform caused nonce reuse in Bitcoin wallets, resulting in large-scale theft of funds [39].

In addition, digital signature algorithms that heavily rely on random numbers may also be vulnerable to replay attacks. A replay attack refers to a scenario where an adversary intercepts a valid digital signature during communication, stores it, and subsequently resends it in a different context or at a later time. This allows the attacker to impersonate a legitimate user or execute unauthorized operations, deceiving the recipient into accepting the request as authentic and current. If the system lacks effective mechanisms to verify the uniqueness or timeliness of a signature, the attacker can repeatedly exploit the same signature, leading to unauthorized access, resource exhaustion, and other serious security threats. Therefore, the degree of reliance on randomness in a digital signature algorithm plays a critical role in determining its overall security, particularly in terms of resisting private key leakage and replay attacks.

To evaluate the security of digital signature algorithms under scenarios involving the reuse of the random number *k*, this study conducts multiple signatures on the same dataset using the same private key and identical random number *k*. This setup simulates scenarios in which an attacker intercepts signature communications or obtains previously signed messages and exploits the reuse of the random number *k* to attempt private key recovery or perform replay attacks. Such attacks may occur in real-world applications due to improper implementation of random number generators or compromise of signature devices. A comparative analysis is then performed to assess the resilience of ECDSA, SM2, and the improved T_SM2 algorithm against such attacks. The experimental results are summarized in Table 6.

#### 5.2.3. Timestamp Precision Analysis

Numerous researchers have proposed various digital signature schemes based on timestamps, either to enable time-based authentication or to enhance the randomness in the signature generation process. In the T_SM2 algorithm proposed in this study, a high-precision timestamp is introduced from an external high-resolution clock. Due to limitations in the local computer hardware, a GNSS common-view receiver is employed to achieve nanosecond-level accuracy. In contrast, most related works rely on UNIX timestamps [20,21], obtained from standard computer hardware, which offer only second-level precision—a significant difference in terms of orders of magnitude.

This substantial improvement in timestamp precision greatly enhances the accuracy of time authentication, allowing the signature generation time to be recorded with much higher accuracy. As a result, the reliability of time validity verification is significantly improved, which is particularly beneficial in time-sensitive fields such as metrology. Furthermore, the nanosecond-level timestamp offers finer-grained temporal input, enriching the entropy of the random seed used during signature generation. This, in turn, strengthens the overall randomness of the signature and its resistance to replay attacks.

By comparison, second-level timestamps, due to their limited resolution, are more likely to cause time window overlaps, which not only reduces the accuracy of time authentication but may also weaken the cryptographic strength of the signature due to insufficient randomness.

#### 5.2.4. Other Security Analyses

The hash function employed by T_SM2 is SM3, which is part of the national cryptographic standard system, offering robust security and compatibility. SM3 is optimized based on SHA-256, with the same output length of 256 bits. SM3 exhibits comparable security strength to SHA-256 in collision resistance, with equivalent computational efficiency. Theoretically, its collision resistance achieves a security level of $2^{128}$ due to the birthday bound, making it suitable for high-security applications. As shown in Table 7, even a minor change of just one bit in the input leads to a significant alteration in the generated hash value, highlighting its high sensitivity to input variations.

T_SM2 is built upon the national cryptographic standard SM2 digital signature algorithm, providing an equivalent level of key security. The private key is generated and securely stored within the cryptographic chip embedded in the user’s local device, ensuring both the authenticity and confidentiality of the key.

Furthermore, to prove that the T_SM2 signature scheme achieves existential unforgeability under adaptive chosen message attacks (EUF-CMA), this paper conducts the analysis based on the following security assumptions:

- The SM3 hash function is collision-resistant;
- The ECDLP is computationally hard to solve.

In the security proof, we introduce a game-based model involving two parties: the challenger *C* and the adversary *A*. The interaction between them is defined as follows:

1. The challenger *C* runs the key generation algorithm of T_SM2 to obtain a private key *d* and computes the corresponding public key *P* for the adversary *A*;
2. The adversary *A* adaptively submits a sequence of messages *M_1_, …, M_n_* to *C*, who returns valid signature pairs $\sigma_{i}\;=\;(r_{i},\;s_{i})$ for each message, following the signing procedure defined in Algorithm 1;
3. Finally, the adversary outputs a new message–signature pair $\left(M^{*},\sigma^{*}\right)=\;\left(M^{*},\left(r^{*},s^{*}\right)\right)$, where $M^{*}\notin \left\{M_{1},\ldots ,M_{n}\right\}$, and attempts to forge a valid signature on $M^{*}$.

If the verification algorithm returns Accept, i.e., $V\mathrm{e}ify\left(M^{*},\sigma^{*},P\right)=Accept$, then the adversary is considered to have succeeded in the attack.

Next, if there exists an adversary *A* that can successfully forge a valid signature pair $\left(r^{*},s^{*}\right)$ with non-negligible probability, then one can construct an algorithm to solve the ECDLP, which contradicts the assumption of its computational intractability. It is important to note that although the adversary may obtain the perturbed timestamp hash values *T_x_* and *T_y_*, these values primarily serve to prevent signature replay, thereby ensuring the uniqueness of each signature. The adversary cannot deduce the ephemeral value *k* or any other sensitive information from *T_x_* and *T_y_*. Therefore, without knowledge of the legitimate private key d, the adversary cannot generate a new valid signature, even with access to the timestamp hashes.

Assuming the adversary successfully forges a signature $\left(r^{*},s^{*}\right)$, the signature must satisfy the following conditions:

(9) $$R=\left(h+x_{1}^{*}+T_{y}^{*}\right)\mathrm{mod}n=r^{*}$$

To achieve this goal, the adversary would need to predict or invert the values of h, $T_{y}^{*}$, and $x_{1}^{*}$, which requires solving the ECDLP or finding a hash collision. As both tasks are computationally infeasible, signature forgery remains impractical.

Therefore, we conclude that the T_SM2 signature scheme is secure under the EUF-CMA model; that is, no efficient adversary can forge a new valid signature under reasonable computational constraints.

### 5.3. Performance Analysis

#### 5.3.1. Elliptic Curve Parameter Performance Analysis

Various elliptic curve parameters have been recommended by scholars internationally, while the T_SM2 algorithm adopts the elliptic curve parameters specified in the SM2 national cryptographic standard. To compare the performance of different elliptic curves under the T_SM2 algorithm, this study selects a dataset of approximately 500KB as the original test data and conducts experiments using five commonly used 256-bit elliptic curves: SM2, secp256k1, secp256r1 (NIST P-256), brainpoolP256r1, and brainpoolP256t1. The testing process includes both signature generation and verification, aiming to evaluate the performance differences of the T_SM2 algorithm when using different elliptic curve parameters.

**Figure 5:**
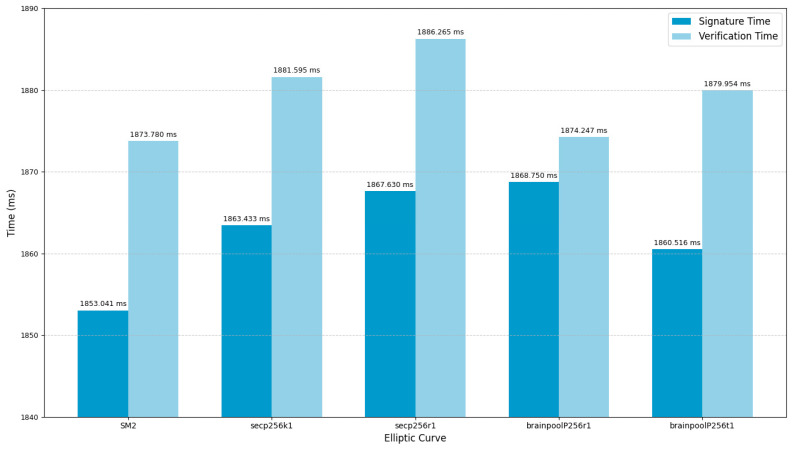
Performance analysis of T_SM2 on different curves.

To ensure the accuracy of the test results and minimize the impact of external factors such as system load, each elliptic curve was tested with 100 iterations of both signature generation and verification. The average execution time was taken as the final performance evaluation metric. As shown in Figure 5, the SM2 curve achieved the shortest execution times in both signing and verification, outperforming the other elliptic curve parameters. These results indicate that the SM2 curve exhibits better compatibility with the T_SM2 digital signature algorithm and delivers the best overall performance.

#### 5.3.2. Data Processing Performance Analysis

To evaluate the performance differences between the T_SM2 digital signature algorithm and the standard SM2 algorithm in processing electronic document data within the metrology domain, a dataset consisting of 20 electronic documents was constructed. The sizes of these files range from several dozen kilobytes to several thousand kilobytes, and the types include plain text files, documents containing tables and images, as well as files with more complex data structures (see Table 8).

**Figure 6:**
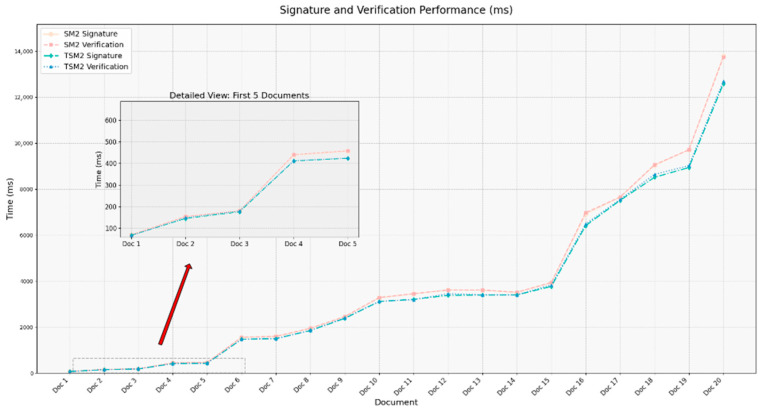
A comparison of execution times for digital signature algorithms.

In the experiment, each document was individually subjected to signature generation and verification using both the T_SM2 and standard SM2 algorithms. To minimize the influence of randomness, each operation was repeated 100 times, and the average value was taken as the performance evaluation metric. The experimental results, shown in Figure 6, indicate that the T_SM2 algorithm consistently outperforms the standard SM2 in terms of average signing and verification times across all documents. The performance advantage of T_SM2 is particularly pronounced when processing large and structurally complex documents. This improvement is mainly attributed to its ability to maintain high randomness and temporal authentication functionality while effectively reducing overall execution time through optimizations such as the elimination of modular inversion operations.

#### 5.3.3. Computational Cost Analysis

In different elliptic curve-based digital signature algorithms, the computational costs of various modules involved in the signature generation and verification phases vary. Drawing on the work of Zhong et al. [40], the computation times for different operations are defined as follows: *T_a_* denotes the computation time for modular addition, *T_m_* for modular multiplication, *T_em_* for elliptic curve point multiplication, *T_ea_* for elliptic curve point addition, and *T_in_* for modular inversion. According to the literature, the computational costs of these operations relative to modular multiplication time *T_m_* are: $T_{em}=29T_{m}$, $T_{ea}=0.12T_{m}$, and $T_{in}=11.6T_{m}$. Since the cost of modular addition *T_a_* is significantly smaller than that of modular multiplication *T_m_*, it is generally considered negligible in cost analysis.

Table 9 presents the computational costs of ECDSA, standard SM2, other schemes from the literature, and the proposed T_SM2 algorithm. It can be observed that T_SM2 achieves superior computational efficiency by avoiding the high-cost modular inversion operation. In particular, it significantly reduces the computational overhead during the signature generation phase, demonstrating improved overall performance compared to other schemes.

## 6. Conclusions

This paper addresses the issues of insufficient computational efficiency and lack of temporal traceability in traditional digital signature algorithms within high-trust metrology applications. An improved digital signature algorithm, T_SM2, derived from SM2, is proposed. This algorithm significantly enhances the temporal consistency and non-repudiation of signature data by introducing nanosecond-level timestamps generated from a trusted time source. Additionally, the algorithm improves computational efficiency by eliminating modular inversion operations and optimizing the signature process structure, without compromising security.

Specifically, the experimental results validate the algorithm’s security advantages: T_SM2 provides complete resistance against attacks such as random number reuse and replay attacks. The introduction of nanosecond-level time information significantly enhances the time-binding mechanism, offering greater security and accuracy compared to traditional methods relying on low-precision UNIX timestamps. In terms of performance, T_SM2 demonstrated a noticeable advantage in both signature generation and verification times when tested on a dataset of 20 electronic documents. By avoiding modular inversion operations, T_SM2 reduced the overall computational cost to 88.12Tm, positioning it at the forefront compared to other improved algorithms.

These experimental findings confirm the effectiveness of T_SM2 in real-world security-critical metrological scenarios, meeting the dual requirements of real-time performance and security in large-scale metrology data processing environments. The nanosecond-level timestamp not only enhances data traceability but also improves the authenticity of time-sensitive data. Overall, T_SM2 demonstrates excellent effectiveness and feasibility in improving signature efficiency, enhancing time-binding capabilities, and ensuring data integrity.

## Figures and Tables

**Table 1:** SM2 elliptic curve parameters.

Curve Parameter	Value
*P*	0xFFFFFFFEFFFFFFFFFFFFFFFFFFFFFFFFFFFFFFFF00000000FFFFFFFFFFFFFFFF
*a*	0xFFFFFFFEFFFFFFFFFFFFFFFFFFFFFFFFFFFFFFFF00000000FFFFFFFFFFFFFFFC
*b*	0x28E9FA9E9D9F5E344D5A9E4BCF6509A7F39789F515AB8F92DDBCBD414D940E93
*G* *_x_*	0x32C4AE2C1F1981195F9904466A39C9948FE30BBFF2660BE1715A4589334C74C7
*G* *_y_*	0xBC3736A2F4F6779C59BDCEE36B692153D0A9877CC62A474002DF32E52139F0A0
*n*	0xFFFFFFFEFFFFFFFFFFFFFFFFFFFFFFFF7203DF6B21C6052B53BBF40939D54123

**Table 2:** Algorithm symbols.

Symbol	Description
*d*	Private key
*P*	Public key
*M*	Message data
$\bar{M}$	Preprocessed message
*ID*	Identifier
*Z*	Preprocessed identifier
*h*	Hash value
*T*	High-precision timestamp
*sig*	Signature
*T_u_*	UTC time (second-level precision)

**Table 3:** DNA encoding rules.

Rule	1	2	3	4	5	6	7	8
00	A	A	T	T	C	C	G	G
01	C	G	C	G	A	T	A	T
10	G	C	G	C	T	A	T	A
11	T	T	A	A	G	G	C	C

**Table 4:** Experimental conditions.

System	CPU	RAM	Software	Timestamp Generation Device
Win11	Intel Core i7-136500HX	16 GB	PyCharm professional 2024 (JetBrains s.r.o., Prague, Czech Republic)	GNSS satellite co-viewing receiver (Star Navigation Space-Time Technology Co., Ltd., Beijing, China)

**Table 5:** Comparative analysis of digital signature algorithms.

Algorithm	Math Basis	Key Length (Equivalent Security)	Signature Length (bytes)	Common Hash Function	User Identity Binding	Timestamp Authentication	Weak Randomness Dependency
RSA	Integer Factorization	3072 bits	384 bytes	SHA-256	×	×	×
ECDSA	ECDLP	256 bits	64 bytes	SHA-256	×	×	×
SM2	ECDLP	256 bits	64 bytes	SM3	√	×	×
T_SM2	ECDLP	256 bits	64 bytes	SM3	√	√	√

**Table 6:** Impact of random k reuse on signature algorithm security.

Algorithm	d	k	sig_1_	sig_2_	K Reuse Resistance	Replay Attack Resistance
ECDSA	57129200…501846	39999300…399137	(0190b8af72ed…6a4b57, 0e75a19d0a68.…e42875)	(0190b8af72ed…6a4b57, aa2e1da311fb…1af81a9)	×	×
SM2	57129200…501846	39999300…399137	(1c3e728cf12b…d74f6b, e176d4f85948…647c59)	(54944e8d54d5…22f2fd, a412f28b62e8…2f95a0)	×	×
T_SM2	57129200…501846	39999300…399137	random	random	√	√

**Table 7:** Analysis of SM3 resistance to hash collision attacks.

Data	Hash Value
1749026124	b5576e7db42b8de544ca27f79fa5018d3d72178835c675091578d9eefb83e4a8
1749026125	c21a58756626b71e457682d49a0e89a7f141eaa66090f9582b501c81b2212301

**Table 8:** Electronic document dataset.

Index	Size (KB)	Category
1	17	Text Documents (≤500 KB)
2	37	
3	45	
4	111	
5	114	
6	384	
7	398	
8	492	
9	622	Documents with Tables and Images (500–1000 KB)
10	828	
11	850	
12	901	
13	905	
14	907	
15	1011	Documents with Complex Data Structures (>1000 KB)
16	1708	
17	1974	
18	2282	
19	2387	
20	3363	

**Table 9:** Computation cost analysis.

Algorithm	Signature Generation	Signature Verification	Total Cost
ECDSA	$T_{em}+T_{in}+T_{m}=41.6T_{m}$	$2T_{em}+T_{in}+{2T}_{m}=71.72T_{m}$	$113.32T_{m}$
SM2	$T_{em}+T_{in}+T_{m}=41.6T_{m}$	$2T_{em}+T_{ea}=58.12T_{m}$	$99.72T_{m}$
Yang et al. [19]	$T_{em}+T_{m}=30T_{m}$	$2T_{em}+T_{ea}=58.12T_{m}$	$88.12T_{m}$
Shao et al. [21]	$T_{em}+T_{in}+T_{m}=41.6T_{m}$	$2T_{em}+T_{ea}=58.12T_{m}$	$99.72T_{m}$
Puthiyidam et al. [20]	$2T_{em}+T_{m}=59T_{m}$	$2T_{em}+T_{ea}=58.12T_{m}$	$117.72T_{m}$
Meng et al. [25]	$2T_{em}+T_{in}+T_{m}=41.6T_{m}$	$3T_{em}+T_{ea}+T_{in}=100.72T_{m}$	$142.32T_{m}$
T_SM2	$T_{em}+T_{m}=30T_{m}$	$2T_{em}+T_{ea}=58.12T_{m}$	$88.12T_{m}$

## Data Availability

The data supporting the reported results in this study are not publicly available due to privacy or ethical restrictions. Further information on the data can be obtained from the corresponding author upon reasonable request.

## References

[1] Rab S., Wan M., Sharma R.K., Kumar L., Zafer A., Saeed K., Yadav S. (2023). Digital Avatar of Metrology. Mapan.

[2] Eichstädt S., Keidel A., Tesch J. (2021). Metrology for the Digital Age. Meas. Sens..

[3] Xiong X., Zhu Y., Li J., Duan Y., Fang X. (2021). A Digital Framework for Metrological Information. Meas. Sens..

[4] Kuster M. (2021). Metrological Data Completeness for Digital Transformation. Proceedings of the 2021 IEEE International Workshop on Metrology for Industry 4.0 & IoT (MetroInd4. 0&IoT).

[5] Abouhogail R.A. (2025). Security of Metrology in the Digital Age: Challenges and Proposed Solutions. Advanced Research Trends in Sustainable Solutions, Data Analytics, and Security.

[6] Toro F.G., Lehmann H. (2021). Brief Overview of the Future of Metrology. Meas. Sens..

[7] Barbosa C.R.H., Sousa M.C., Almeida M.F.L., Calili R.F. (2022). Smart Manufacturing and Digitalization of Metrology: A Systematic Literature Review and a Research Agenda. Sensors.

[8] Gadelrab M.S., Abouhogail R.A. (2021). Towards a New Generation of Digital Calibration Certificate: Analysis and Survey. Measurement.

[9] Mustapää T. (2022). Digitalisation of Metrology for Improving Trustworthiness and Management of Measurement Data in Industrial IoT Systems. Ph.D. Thesis.

[10] De Santis L. (2022). Blockchain: The Distributed Paradigm for Secure Metrology Digitalization.

[11] Penubadi H.R., Shah P., Sekhar R., Alrasheedy M.N., Niu Y., Radhi A.D., Tharwat M., Tawfeq J.F., Gheni H.M., Abdulbaqi A.S. (2023). Sustainable Electronic Document Security: A Comprehensive Framework Integrating Encryption, Digital Signature and Watermarking Algorithms. Herit. Sustain. Dev..

[12] Shankar G., Ai-Farhani L.H., Anitha Christy Angelin P., Singh P., Alqahtani A., Singh A., Kaur G., Samori I.A. (2023). Improved Multisignature Scheme for Authenticity of Digital Document in Digital Forensics Using Edward-curve Digital Signature Algorithm. Secur. Commun. Netw..

[13] Teng D., Yao Y., Wang Y., Zhou L., Huang C. (2022). An Sm2-Based Traceable Ring Signature Scheme for Smart Grid Privacy Protection. Proceedings of the International Conference on Wireless Algorithms, Systems, and Applications.

[14] Yang M., Liu C., Li H., Shao C. (2022). Efficient SM2 Hardware Design for Digital Signature of Internet of Vehicles. Proceedings of the 2022 IEEE International Conference on Trust, Security and Privacy in Computing and Communications (TrustCom).

[15] Li J., Liu Y., Li S., Zhang G., Gao X., Gong P. (2023). Self-C2AD: Enhancing CA Auditing in IoT with Self-Enforcement Based on an SM2 Signature Algorithm. Mathematics.

[16] Ouyang S., Liu X., Liu L., Wang S., Shao B., Zhao Y. (2024). An Efficient and Provably Secure SM2 Key-Insulated Signature Scheme for Industrial Internet of Things. CMES-Comput. Model. Eng. Sci..

[17] Shao C., Li W., Li H., Tang Z., Liang J. (2025). A Novel Lattice-Based Fault Injection Attack Targeting the Nonce in the SM2 Digital Signature Algorithm. ACM Trans. Embed. Comput. Syst..

[18] Sethi P.C., Sahu N., Behera P.K. (2022). Group Security Using ECC. Int. J. Inf. Technol..

[19] Yang X., Liu Y., Wu J., Han G., Liu Y., Xi X. (2021). Nomop-Ecdsa: A Lightweight Ecdsa Engine for Internet of Things. Wirel. Pers. Commun..

[20] Puthiyidam J.J., Joseph S., Bhushan B. (2024). Temporal ECDSA: A Timestamp and Signature Mask Enabled ECDSA Algorithm for Iot Client Node Authentication. Comput. Commun..

[21] Shao Y., Wang Y., Yang Y., Wang X. (2022). Research on a Secure Communication Protocol Based on National Secret SM2 Algorithm. J. Comput. Commun..

[22] Wu K., Cheng R., Cui W., Li W. (2021). A Lightweight SM2-Based Security Authentication Scheme for Smart Grids. Alex. Eng. J..

[23] Chen F., Liu Y., Zhang T., Xie D., Shen Z. (2022). SM2-Based Low-Cost and Efficient Parallel Modular Multiplication. Microprocess. Microsyst..

[24] Xu S., Deng Y., Tian Y., Liu C., Liu R. (2023). Design and Implementation of a Cloud-Based Collaborative Signature System for Two Parties Based on SM2. Proceedings of the 2023 3rd International Symposium on Computer Technology and Information Science (ISCTIS).

[25] Meng C., Meng L., Liang S. Research on New Encryption Technology Based on SM2 Asymmetry. Proceedings of the Proceedings of the 2024 International Conference on Intelligent Education and Computer Technology.

[26] Zhu H., Li D., Sun Y., Chen Q., Tian Z., Song Y. (2024). Optimization of SM2 Algorithm Based on Polynomial Segmentation and Parallel Computing. Electronics.

[27] Koblitz N. (1987). Elliptic Curve Cryptosystems. Math. Comput..

[28] Miller V.S. (1985). Use of Elliptic Curves in Cryptography. Proceedings of the Conference on the Theory and Application of Cryptographic Techniques.

[29] Genç Y., Afacan E. (2021). Design and Implementation of an Efficient Elliptic Curve Digital Signature Algorithm (ECDSA). Proceedings of the 2021 IEEE International IOT, Electronics and Mechatronics Conference (IEMTRONICS).

[30] Sadkhan S.B. (2021). Development of Solving the ECDLP. Proceedings of the 2021 7th International Engineering Conference “Research & Innovation amid Global Pandemic”(IEC).

[31] Johnson D., Menezes A., Vanstone S. (2001). The Elliptic Curve Digital Signature Algorithm (ECDSA). Int. J. Inf. Secur..

[32] Ulla M.M., Khan M.S., Sakkari D.S. (2023). Implementation of Elliptic Curve Cryptosystem with Bitcoin Curves on SECP256k1, NIST256p, NIST521p, and LLL. J. ICT Stand..

[33] ISO/IEC JTC 1/SC 27 ISO/IEC 14888-3:2018/CD Amd 1—IT Security Techniques—Digital Signatures with Appendix—Part 3: Discrete Logarithm Based Mechanisms—Amendment 1. https://www.iso.org/standard/89517.html.

[34] Zellagui A., Naima H.-S.A.A. (2022). A Comparative Study Between the Standard Hash Function Sha-2 and the Chinese Standard Hash Function SM3. Proceedings of the 4th International Baku Scientific Research Congress.

[35] Jia Z., Wang Y., Xu H., Xu Q., Yang D. (2024). Long-Term Stability of GNSS Receiver Delays in Time and Frequency Comparisons. Proceedings of the 2024 Conference on Precision Electromagnetic Measurements (CPEM).

[36] Verma A.K., Dave M., Joshi R.C. (2008). DNA Cryptography: A Novel Paradigm for Secure Routing in Mobile Ad Hoc Networks (MANETs). J. Discret. Math. Sci. Cryptogr..

[37] Watson J.D., Crick F.H.C. (1953). Molecular Structure of Nucleic Acids. Nature.

[38] Hotz G. Console Hacking 2010-Ps3 Epic Fail. Proceedings of the 27th Chaos Communications Congress.

[39] Bitcoin Project Android Security Vulnerability. https://bitcoin.org/en/alert/2013-08-11-android.

[40] Zhong X., Guanzhong D., Deming Y. (2006). An Efficient ECDSA-Based Signature Scheme for Wireless Networks. Wuhan Univ. J. Nat. Sci..

